# Decomposition Analysis of Socioeconomic Inequalities in Vaccination Dropout in Remote and Underserved Settings in Ethiopia

**DOI:** 10.4269/ajtmh.23-0816

**Published:** 2024-06-04

**Authors:** Fisseha Shiferie, Samson Gebremedhin, Gashaw Andargie, Dawit A. Tsegaye, Wondwossen A. Alemayehu, Teferi Gedif Fenta

**Affiliations:** ^1^ Project HOPE Ethiopia Country Office, Addis Ababa, Ethiopia;; ^2^ School of Pharmacy, Addis Ababa University, Addis Ababa, Ethiopia;; ^3^ School of Public Health, Addis Ababa University, Addis Ababa, Ethiopia;; ^4^ Project HOPE Headquarters, Washington, District of Columbia

## Abstract

Despite increments in immunization coverage over the past decades, substantial inequality due to wealth status has persisted in Ethiopia. This study aimed to decompose the concentration index into the contributions of individual factors to socioeconomic inequalities of childhood vaccination dropout in remote and underserved settings in Ethiopia by using a decomposition approach. A wealth index was developed by reducing 41 variables related to women’s household living standards into nine factors by using principal component analysis. The components were further totaled into a composite score and divided into five quintiles (poorest, poorer, middle, richer, and richest). Vaccination dropout was calculated as the proportion of children who did not get the pentavalent-3 vaccine among those who received the pentavalent-1 vaccine. The concentration index was used to estimate socioeconomic inequalities in childhood vaccination dropout, which was then decomposed to examine the factors contributing to socioeconomic inequalities in vaccination dropout. The overall concentration index was −0.179 (*P* <0.01), confirming the concentration of vaccination dropout among the lowest wealth strata. The decomposition analyses showed that wealth index significantly contributed to inequalities in vaccination dropout (49.7%). Place of residence also explained −16.2% of the inequality. Skilled birth attendance and availability of a health facility in the kebele (the lowest administrative government structure) also significantly contributed (33.6% and 12.6%, respectively) to inequalities in vaccination dropout. Wealth index, place of residence, skilled birth attendance, and availability of a health facility in the kebele largely contributed to the concentration of vaccination dropout among the lowest wealth strata. Policymakers should address vaccination inequality by designing more effective strategies.

## INTRODUCTION

In spite of the remarkable global improvement in basic vaccination coverage, there are inequalities in access to childhood vaccination within and between countries. Within countries, child vaccination data showed that richer subgroups tend to have higher coverage, whereas the coverage among poorer subgroups varied across countries.^1^ For example, studies in India,^2^ Nigeria,^3^ and Brazil^4^ indicated that children of mothers who had higher education levels and household wealth status were more likely to have high vaccination coverage. Studies from low- and middle-income countries suggested that immunization coverage is pro-rich in most countries. Gambia, Namibia, and Kyrgyz Republic were the only countries where children who belonged to higher socioeconomic status group were less likely than their lower socioeconomic status counterparts to receive all four core vaccines.^5^^,^^6^

Despite the steady increments in immunization coverage over the past decades, pro-urban, pro-rich, and pro-educated inequalities in vaccination coverage have been observed in sub-Saharan African countries.^7^^–^^9^ West African countries such as Guinea, Mali, and Nigeria had the widest equity gaps in immunization, with inequalities in cover ages and dropouts mostly related to poverty, low maternal education, and living in certain disadvantaged subnational regions. In these countries, inequalities in bacillus Calmette-Guérin (BCG) and diphtheria, tetanus, and pertussis three (DTP3) coverage and dropouts were greater than 20 percentage points between the wealthiest and the poorest, between high-coverage regions and low-coverage regions, and between children of mothers with at least secondary education and those with no formal education. Place of residence contributed minimally to the inequality in coverage and dropout.^10^ A study conducted in 24 countries in the African region revealed that the difference in vaccination dropout estimates between the highest and lowest quintiles was 14.9% or more. These inequalities in dropout rate stemmed from lower estimated national immunization coverages.^11^

According to the Ethiopian Demographic and Health Surveys (DHS) of 2011 and 2016, the uptake of BCG, DTP3, oral polio vaccine three (OPV3), and measles vaccines was disproportionately concentrated among children from wealthy households. For example, in 2016, DTP3 had a concentration index (CnI) of 0.175. The estimate for the distribution of children who received no vaccination increased from a CnI of −0.092 in 2011 to a CnI of −0.184 in 2016. The negative values for children who received no vaccination confirms pro-poor distributions. The decomposition results showed that the significant contributors to socioeconomic inequality in basic vaccination status included wealth index, maternal education, contraceptive use, antenatal care (ANC) contacts, exposure to media, and place of residence. The use of maternal health services had the highest significant contributions to socioeconomic inequalities in child vaccination. Antenatal care contacts had a 45.4% contribution in 2011 and a 50.4% contribution in 2016. Wealth status is the other significant contributor, 23.9% in 2011 and 21.2% in 2016. On the other hand, rural residence had a negative contribution to socioeconomic inequalities in child vaccination in both surveys.^12^^–^^14^

Although the establishment of more community-level service delivery points over the last two decades and the provision of free vaccination services at public health facilities improved childhood vaccination coverage, substantial inequality due to wealth status has persisted in Ethiopia.^15^^–^^17^ Another study conducted in Ethiopia indicated the presence of persistent socioeconomic inequalities in terms of vaccine uptake. Children from the poorest households lagged behind those from relatively wealthy households in vaccination. A decomposition analysis of socioeconomic inequalities revealed that maternal educational level, ANC use, institutional delivery, and exposure to media consistently contributed to the inequality observed in vaccine uptake.^18^

Many studies in Ethiopia have assessed vaccination coverage, predictors associated with vaccination coverage, and socioeconomic inequalities in vaccination coverage. However, there has been no study that attempted the decomposition of socioeconomic inequalities in vaccination dropout especially in remote and underserved settings (including pastoralist regions, developing regions, newly established regions, conflict-affected areas, underserved urban populations, internally displaced populations [IDPs], and refugee populations) of Ethiopia. Hence, the objective of this study was to decompose the CnI into the contributions of individual factors to socioeconomic inequalities of childhood vaccination dropout by using a decomposition approach.

## MATERIALS AND METHODS

### Study design and settings.

This study was part of a cross-sectional national evaluation survey that was conducted from May to July 2022. Prior to the quantitative survey, zero-dose and underimmunized areas were identified through qualitative situational and geospatial analyses based on secondary data sources. The study was conducted by following a single-round, cross-sectional survey design.

In line with the understanding that nearly half of zero-dose and under-immunized children in low-income countries are from hard-to-reach communities, conflict-affected settings, or disadvantaged urban areas,^19^ this study targeted populations in the following eight partly overlapping settings:

1. Pastoralist regions and populations: Afar and Somali regions and specific pastoralist or semipastoralist settings in Oromia, Southern Nations, Nationalities, and Peoples (SNNP), Southwest Ethiopia Peoples, and Gambella regions.
2. Developing regions: Afar, Somali, Gambella, and Benishangul Gumuz regions.
3. Newly established regions: Sidama and Southwest Ethiopia Peoples regions.
4. Conflict-affected areas: selected settings in Afar, Amhara, Oromia, and Benishangul Gumuz regions.
5. Underserved urban populations: urban slums in six selected cities (Addis Ababa, Bahirdar, Hawassa, Dire Dawa, Harar, and Adama) and rural areas under Dire Dawa City administration and the Harari region.
6. Hard-to-reach areas in major regions: selected remote districts in the Amhara, Oromia, and SNNP regions.
7. Internally displaced persons: selected IDP centers in the Afar, Amhara, Oromia, and Benishangul Gumuz regions.
8. Refugees: refugees from selected camps in the Somali, Afar, and Gambella regions.^20^^,^^21^

### Study participants.

The target population was children under 5 years old who resided in underserved, remote, and conflict-affected areas of Ethiopia. All children aged 12 to 35 months were included as study participants.

### Sample size determination.

The sampling design of the study was adopted from WHO’s 2018 *Vaccination Coverage Cluster Surveys* manual.^22^ The adequate sample size for each of the target populations was calculated using Cochran’s single proportion sample size formula,^23^ assuming 95% confidence level, 4% margin of error, 16% prevalence of zero-dose children,^13^ and 10% compensation for possible nonresponse. The following sample size formula was used to calculate the number of children required in a prevalence study: $n=\frac{Z2p\left(1-p\right)}{d2}$, where *n* is the total sample size needed, *Z* is the statistic corresponding to the 95% confidence level, which is 1.96, *p* is the prevalence of zero-dose children in previous studies in Ethiopia, and *d* is precision (corresponding to effect size).^24^ Therefore, a sample size of 360 was required for each of the aforementioned target population domains.

According to the Ethiopian DHS 2016 and Mini DHS 2019 data, an average of 12 children aged 12 to 35 months of age are available per enumeration area (EA).^13^^,^^25^ Therefore, for each of the population domains, a minimum of 30 EAs were required to recruit 360 children in each target population, assuming all children in the EA would be eligible for inclusion in the study. In urban slums, 40 EAs were randomly selected, and 480 children were selected (Table 1).^20^^,^^21^

**Table 1:** Total sample size and EAs required for the vaccination coverage survey, Ethiopia, 2022

Types of Study Population	Number of EAs	Total Sample Size (Number of Children)
Pastoralist areas in Oromia, SNNP, and Southwest Ethiopia Peoples regions	30	360
Hard-to-reach areas	30	360
Conflict-affected areas	30	360
Refugees	30	360
IDPs	30	360
Newly formed regions: Sidama and Southwest Ethiopia Peoples regions	30	360
Urban slums	40	480
Afar	30	360
Somali	30	360
Benishangul Gumuz	30	360
Gambella	30	360
Total	340	4,080

EAs = enumeration areas; IDPs = internally displaced persons; SNNP = Southern Nations, Nationalities, and Peoples.

Initially, it was planned to include 4,080 children from 340 EAs (a minimum of 360 sample per population domain) in the survey. However, in the actual survey, 3,646 children aged 12 to 35 months from 340 EAs were enrolled because of conflict in some of the study districts. The total sample size was large enough to allow subgroup inequality analysis based on sex, age, and other pertinent background characteristics, including socioeconomic status.^20^^,^^21^

### Sampling procedure.

Children aged 12 to 35 months were selected using a cluster sampling approach in a two-step procedure. First, EAs were randomly selected from the total EAs available in each target population domain.^20^^,^^21^ In this case, the EAs delineated by the Central Statistical Agency of Ethiopia for the recent census were used as a sampling frame.^13^ In the case of urban slums, hotspot urban slums in Addis Ababa, Adama, Bahir Dar, Hawassa, Harar, and Dire Dawa cities were located and delineated, and EA maps were drawn by experienced cartographers. In the case of IDP and refugee camps, villages or clusters were considered EAs. Second, all eligible children in each EA were listed, and 12 children were ultimately selected using a smartphone-based random-number generator.^20^^,^^21^

### Data collection procedures and data quality assurance.

Data were collected using pretested tools prepared in five local languages (Amharic, Afan Oromo, Somali, Afar, and Sidama). Survey data were collected by 48 experienced enumerators and 24 supervisors using the CommCare digital app, an open-source and user-friendly application system which is interoperable with major data analytics and visualization software. The app helped to collect individual child-level and household information to ensure high-quality data collection, cleaning, and monitoring in real time.^20^^,^^21^

Enumerators and supervisors were recruited based on educational status (at least diploma holders in a health-related discipline), previous experience in similar national surveys, and familiarity with the CommCare digital app.^21^ Prior to deployment, the enumerators and supervisors received a 5-day training guided by a structured training manual. The training included an explanation of the sampling approach, basic principles of data collection, line-by-line discussion on the questionnaire, an overview on using the CommCare digital app, mock interviews, field practice, and a review of basic ethical practices of research involving human participants.^20^

Data collectors were allowed to collect data from up to six individuals per person per day. To validate the quality of the data, one-third of all the study participants were reinterviewed by the supervisors. The uploaded data were closely monitored by the research team throughout the survey implementation period.^20^

### Ascertainment of childhood vaccination.

The vaccination status of children was ascertained based on three different sources of information, including caregiver reports, home-based (vaccination card) reports, and facility-based reports, as recommended by the WHO. In areas where the mother or caregiver presented an immunization card, the child’s immunization status was based on vaccination card review. Where an immunization card was not available, the immunization status was assessed according to mothers’/caregivers’ self-reports/recalls. Facility-based reports were considered in determining vaccination status when a mother/caregiver reported that her child was vaccinated but a vaccination card was not available. In this case, data collectors were expected to visit the nearby health facility to verify mother’s/caregiver’s self-report/recall based on the available medical record. Previous studies have proven this to be a reliable method to ascertain childhood vaccination in resource-limited settings with poor documentation of childhood immunization.^21^^,^^26^

### Variables of the study.

Vaccination dropout is the proportion of children who did not receive the subsequent vaccine among those who received the first vaccine. It was calculated as the proportion of children who did not receive the pentavalent-3 vaccine (the third dose that is given to a child at 14 weeks of age) among those who received the pentavalent-1 vaccine (the first dose that is given at 6 weeks of age). The predictor variables of vaccination dropout include wealth index, marital status, child age, respondent age, time to walk to the health facility (one way), maternal educational status, paternal educational status, caregiver’s employment status, ANC visits, postnatal care (PNC) services, place of residence, number of children under 5 years of age, skilled birth attendance (SBA), availability of a health facility in the kebele (the lowest administrative government structure), gender empowerment, child sex, and sex of the household head.^21^

Gender empowerment is a composite index measuring gender inequality in economic participation, decision-making mainly on health-related matters, and power over economic resources. Gender empowerment was measured using a composite scale (minimum = 0, maximum = 6). Women’s reported power based on six components (major household purchases, expending own income, expending partner’s income, visiting families/relatives, and deciding on health care for herself and the index child) was used to develop the scale. Each component was coded as “1” when the decision was made by the woman or jointly with her partner and “0” when the decision was made solely by the partner. Finally, a score out of 6 was computed and categorized into low (0–2), medium (3 or 4), or high (5 or 6) ordinal categories.^20^^,^^21^

Wealth index is a composite variable that measures the woman’s household living standards. At the design stage, a representative mix of variables considering the contexts of urban and rural communities was carefully selected. Wealth index was computed as a composite index of living standard as recommended by the DHS program. It was developed based on ownership of valuable assets and livestock, size of land for agriculture and housing purposes, materials used for house construction, and access to basic social services, including electricity, banking, improved water sources, and body waste disposal methods. A total of 41 variables were reduced into nine factors using principal component analysis. The components were further totaled into a score and ultimately divided into five quintiles (poorest, poorer, middle, richer, and richest).^13^^,^^20^^,^^21^

## STATISTICAL ANALYSES

Data were collected using the CommCare digital app^27^ and stored in a local server on a daily basis. It was exported to STATA version 17.0^28^ for advanced statistical analysis. To balance weighted and unweighted sample sizes, linearization of poststratification weights were made.^21^

The outcome variable was whether a child had dropped out of the third dose of the pentavalent vaccine. Predictor variables considered in the current study were selected based on proven relationships and predicting factors from published literature and their biological plausibility. These included wealth status, marital status, child age, respondent age, time to walk to health facility (one way), maternal educational status, caregiver’s employment status, ANC visits, PNC services, place of residence, number of children under 5 years of age, SBA, availability of a health facility in the kebele, gender empowerment, child sex, and sex of the household head.^20^^,^^21^

### Vaccination dropout inequality by wealth status.

Vaccination dropout inequality by wealth status was estimated using the CnI. The CnI is described as two times the area between the line of equality and the concentration curve. The index takes a value between −1 and +1; an index of 0 indicates the presence of equality in the uptake of vaccines. If wealth-related inequalities exist, it can be seen in one of two forms. The first is when there is uneven concentration of dropout among the rich, and, in this case, the CnI takes on a positive value. The second is negative-value CnI, which implies a high concentration of vaccination dropout among the poor. The greater the magnitude of inequality, the wider the gap will be between the line of equality and the CnI.^14^^,^^21^

The CnI can then be measured as follows: twice the covariance of the health variable and the ranking of the living standards variable *r* all divided by the mean of the health measure (µ). Greater absolute CnI values indicate greater inequality in vaccination dropout.

$$\text{CnI}=\frac{2}{\mu }cov\;\left(h,r\right)$$ (1)

This study makes use of the Erreygers corrected CnI, which is algebraically expressed as shown below.

$$E\left(h\right)=\frac{4\mu }{b-a}CnI$$ (2)

Where µ is the mean of the health variable (vaccination dropout), *CnI* is the standard CnI, with *b* and *a* representing the upper and lower bounds of the outcome variable (*h*). In our study, the range *b–a* is unity, as the outcome variable is binary.^29^

### Decomposing socioeconomic inequality of vaccination dropout.

The values of CnI show and quantify the level of inequalities related to wealth in the use of health services. However, it does not highlight the pathways through which inequality occurs. Policymakers are also interested in the factors that contribute to wealth-related inequalities in vaccination dropout. The CnI of a health variable can be decomposed into the contributions of individual factors to the overall level of wealth-related inequalities in childhood vaccination dropout. This can be done using an approach developed by Wagstaff et al.^30^

Our health variable *hi* (vaccination dropout) is linked to a set of explanatory variable *Xij* by the following linear model.

$$hi=\alpha +\sum_{j=1}^{q}\left(\beta \text{jXj}\right)+\;\text{εi}$$ (3)

If we have a linear model such as that shown in Equation (3), Wagstaff et al. show that the CnI for *hi* can be written as:

$$\text{CnI}\;\left(h\right)=\sum_{j=1}^{q}\frac{\left(\beta j\text{x}\bar{\text{x}}j\right)}{\mu h}\text{CnI}\;\left(\text{Xj}\right)+\;\frac{GC\varepsilon }{\mu h}$$ (4)

In Equation (4), *CnI (h)* is the CnI for the health variable *h* (vaccination dropout), $\bar{\text{x}}j$ is the mean of *xj*, *µh* is the mean of the health variable, *CnI(xj)* is the CnI for *xj,* and *GCε* is the generalized CnI for the error term. In this equation, the first part is the weighted sum of the CnI for the variable *xj*. The weight of each regressor is determined by the elasticity ($\beta \bar{\text{x}}j$) of *h* with respect to *xj*. The second part of the equation is the residual socioeconomic inequalities in health that cannot be explained by the CnI of the regressors. Since we applied the Erreygers normalization to the calculation of the CnI for the socioeconomic inequalities in vaccination dropout, the corrected CnI for the health variable is formulated as:

$$E\;\left(h\right)=4\sum_{j=1}^{q}\beta \text{jXjCnI}\;\left(\text{Xj}\right)+\;4\text{GC}\varepsilon$$ (5)

Equation (5) can now be used to decompose socioeconomic inequalities in vaccination dropout, showing the contribution of each factor. If the contribution of variable *x* is positive, then inequality in the health variable would decrease if variable *x* becomes equally distributed across the socioeconomic group, ceteris paribus. The opposite is also true, i.e., if a contribution is negative, the absence of inequalities in that variable would result in an increase in inequality, ceteris paribus. The absolute contribution a variable makes to socioeconomic inequality is a product of the elasticity ($\beta \bar{\text{x}}j$) of vaccination dropout for each variable and the CnI for each variable. Therefore, to estimate the contribution, we need to first estimate the coefficients of the explanatory variables via a regression. Ordinary least squares, probit, and generalized linear models are the three most common regression methods used for decomposition of inequalities.^30^^,^^31^ The generalized linear model for decomposition of the Erreygers CnI was used in this study. A bootstrapping technique was used to generate the standard errors for the absolute contributions. Data analysis was conducted in STATA 17, and poststratification sample weights were used in all analyses to adjust for unequal probabilities of selection and nonresponse.^21^

### Ethical approval and consent to participate in the study.

The research was implemented in compliance with national and international ethical principles. The research protocol was reviewed and approved by the institutional review board of the Ethiopian Public Health Institute (416/2021). Information was collected after receiving written informed consent from the caretakers. To maximize beneficence, all zero-dose children were referred to the nearest health facility using a referral form.

## RESULTS

### Sociodemographic characteristics.

A total of 3,646 mothers/caregivers with children aged 12 to 35 months participated in the study with a response rate of 97.7%. Over half of (54%) respondents were between 25 and 34 years of age, and the majority of them (59.2%) had no formal education. More than 81% of respondents were from rural areas, and 17% of them were from the Afar region. Nearly 91% of the respondents were married/living together, and 57% were unemployed at the time of the survey (Table 2).^21^

**Table 2:** Sociodemographic characteristics of respondents and children in underserved settings of Ethiopia, 2022

Characteristics	Frequency (Number of Respondents or Children)	Percent
Child’s sex		
Male	1,985	54.4
Female	1,661	45.6
Child’s age (months)		
12–23	1,849	50.7
24–35	1,797	49.3
Respondent’s age (years)		
15–24	875	24.0
25–34	1,969	54.0
35–44	572	15.7
≥45	104	2.9
Do not know	126	3.5
Respondent’s educational status		
No formal education or preschool	2,158	59.2
Primary education	788	21.6
Secondary education	616	16.9
Tertiary education	84	2.3
Marital status		
Not ever married	43	1.2
Married/living together	3,312	90.8
Separated	83	2.3
Divorced	110	3.0
Widowed	98	2.7
Place of residence		
Urban	677	18.6
Rural	2,969	81.4
Caregiver’s employment status		
Unemployed	2,098	57.6
Employed	1,548	42.4
Region*		
Afar	636	17.4
Amhara	372	10.2
Oromia	431	11.8
Somali	480	13.2
Benishangul Gumuz	216	5.9
Southern Nations, Nationalities, and Peoples	300	8.2
Sidama	239	6.6
Southwest Ethiopia Peoples	181	5.0
Gambella	479	13.1
Harari	60	1.6
Addis Ababa	192	5.3
Dire Dawa	60	1.6
Household size (no. of people)		
2–5	2,044	56.0
≥6	1,602	44.0

* Unweighted sample size.

### Vaccination dropout.

Vaccination dropout was calculated using Pentavalent-1 and Pentavalent-3 vaccines. The overall vaccination dropout estimate was 44%. While urban slums had the lowest dropout estimate (11.2%), the highest dropout estimate was from developing regions (60.1%), followed by IDPs (57.1%) (Table 3).

**Table 3:** Pentavalent-1 to pentavalent-3 vaccination dropout across different remote and underserved population groups of Ethiopia, 2022

Population Group	Pentavalent-1 to Pentavalent-3 Dropout, % (95% CI)
Urban slums	11.2 (8.3–14.7)
Conflict-affected regions	38.6 (32.0–45.9)
IDPs	57.1 (48.9–65.1)
Hard-to-reach in agrarian regions	40.0 (36.1–44.2)
Pastoralist population	49.3 (46.0–52.6)
Developing regions	60.1 (55.9–64.2)
Newly formed regions	48.8 (42.0–55.4)
Refugees	45.4 (38.7–52.3)
All	44.0 (41.9–45.9)

IDPs = internally displaced persons.

### Vaccination dropout inequality by wealth status.

Vaccination dropout by wealth status was also estimated. Children born from mothers/caregivers who were at the lower level of the wealth quintile had the highest dropout rates in comparison with their richest counterparts (59% versus 35%) (Table 4).

**Table 4:** Dropout rate across the five wealth groups in remote and underserved population groups of Ethiopia, 2022

Wealth Status	Dropout Rate, %
Poorest	59.0
Poorer	47.5
Middle	44.0
Richer	36.0
Richest	35.0

The statistically significant negative value of the CnI (−0.179; *P* <0.01) in Table 5 confirms the concentration of vaccination dropout among the lowest wealth strata.^21^

**Table 5:** Concentration index and standard error for the outcome variable

Index	No. of Observations	Index Value	Standard Error	*P*-Value
Erreygers normalized CnI	2,464	−0.17865141	0.02232006	0.0000

CnI = concentration index.

### Decomposing socioeconomic inequality of vaccination dropout.

Before the decomposition analysis was carried out, the multicollinearity among predictor variables was assessed using the variance inflation factor (VIF). The VIF ranged from 1.01 to 2.17, which implied that multicollinearity does not exist among these variables. Several published articles showed that wealth index is a major determinant that influences socioeconomic inequality. Hence, we included wealth index as a predictor variable to quantify the magnitude of wealth-related inequalities in vaccination dropout. Presented in the first column of Table 6 are CnIs of predictor variables. This column provides insights into the distributions of vaccination dropout among the different wealth quintiles and the socioeconomic determinants. The CnI for children from the lowest wealth strata was negative, which indicated that childhood vaccination dropout was concentrated among the relatively poorer families. Furthermore, mothers who had lower educational status, who were residing in rural areas, who were living in areas where it took over 30 minutes to get to the nearest health facility, who were less gender empowered, who had two or more children under 5 years old, and who were in the age range of 25 to 34 years had a negative CnI value, indicating that these variables were concentrated among people of lower socioeconomic (poorer) status.

**Table 6:** Decomposition of socioeconomic inequalities in vaccination dropout in remote and underserved settings of Ethiopia, 2022 (*N* = 2,417)

Variables (*N =* 2,417)	CnI	Elasticity	Margins	% Contribution	Total % Contribution
Wealth status					
Poorest (ref.)					
Poorer	−0.39*	−0.09*	−0.11*	−20.8*	
Middle	−0.02	−0.10	−0.12*	−1.0	
Richer	0.40*	−0.16*	−0.17*	35.8*	
Richest	0.56*	−0.11*	−0.17*	35.7*	49.7
Maternal educational status					
No formal education (ref.)					
Primary education	−0.01	−0.04	−0.04	−0.1	
Secondary education	0.22*	−0.01*	−0.01	0.8*	
Tertiary education	0.08*	−0.02*	−0.18*	1.0*	1.7
Caregiver’s employment status					
Working	0.06^†^	0.15^†^	0.09*	−4.8^†^	−4.8
Not working (ref.)					
Place of residence					
Urban (ref.)					
Rural	−0.19*	−0.14*	−0.05	−16.2*	−16.2
Child age (months)					
12–23 (ref.)					
24–35	0.03	0.14	0.07*	−2.4	−2.4
Number of children under 5 years old					
1 (ref.)					
2	−0.09*	0.19*	0.11*	9.0*	
≥3	−0.03^†^	0.03^†^	0.10^†^	0.4^†^	9.4
Marital status					
Not married (ref.)					
Married	0.03*	0.012*	0.03	2.3*	2.3
Respondent age (years)					
15–24 (ref.)					
25–34	−0.05^†^	0.14^†^	0.06*	3.8^†^	
35-44	0.04^†^	0.04^†^	0.06^†^	−0.9^†^	
≥45	0.01	0.05	0.49*	−0.0	2.9
ANC contacts					
<4 (ref.)					
≥4	0.13*	−0.08*	−0.05^†^	5.7*	5.7
SBA					
No (ref.)					
Yes	0.23*	−0.26*	−0.11*	33.6*	33.6
PNC contacts					
No (ref.)					
Yes	−0.01	0.21	0.10*	0.8	0.8
Availability of health facility in the kebele					
Yes	0.07*	−0.31*	−0.08	12.6*	12.6
No (ref.)					
One-way walking distance to the nearest health facility (minutes)					
≤30 (ref.)					
>30	−0.11*	0.14*	0.07*	8.4*	8.4
Gender empowerment					
Not married (ref.)					
Low	−0.05*	0.05*	0.12*	1.3*	
Medium	0.01	0.01	0.01	−0.0	
High	0.07*	0.0*	0.0	0.0*	1.3
Child sex					
Male (ref.)					
Female	0.04	−0.03	−0.01	0.6	0.6
Sex of household head					
Female	0.04*	−0.40*	−0.11^†^	8.4*	8.4
Male (ref.)					
Total					114

ref. = reference category.

* *P* <0.01.

^†^ *P* <0.05. ref. = reference category.

Estimated marginal effects from the regression analysis are presented in the third column of Table 4. The marginal effects indicated the association between the predictor variables and outcome indicator. Wealth index, maternal educational status, respondent age, time to walk to the nearest health facility, caregivers’ employment status, ANC, PNC, SBA, women’s empowerment, number of children under 5 years old, and child age were significantly associated with vaccination dropout (Table 6).

Table 6 also summarizes the percentage contributions of the predictor variables to socioeconomic-related inequalities in vaccination dropout. Predictor variables that contributed the most to socioeconomic inequalities in vaccination dropout were place of residence (urban or rural), wealth index, SBA, and availability of a health facility in the kebele. The absolute values of the percentage contributions of predictors indicated the magnitude of their contributions to inequality. A positive value for a percentage contribution indicates that the predictor increases the inequality, and the opposite is true for a negative value. Our study showed that wealth index is a significant contributor to inequalities in vaccination dropout (49.7%). Place of residence also explained −16.2% of the inequality in vaccination dropout. SBA and availability of health facility in the kebele were also large contributors to inequality, contributing 33.6% and 12.6% to inequalities in vaccination dropout, respectively.

The negative contribution by place of residence indicated that if place of residence was equally distributed across the wealth index, then inequalities in vaccination dropout would increase by 16.2%. On the other hand, the positive contribution by SBA indicated that if SBA was equally distributed across the wealth index, then inequalities in vaccination dropout would decrease by 33.6%. Furthermore, number of children under 5 years old, sex of the household head, and one-way walking distance to the nearest health facility also contributed 9.4%, 8.4%, and 8.4% to the inequality of vaccination dropout, respectively.

## DISCUSSION

To the best of our knowledge, this is the first study to quantify the socioeconomic inequalities in vaccination dropout and its drivers in Ethiopia. The study decomposed the CnI into the contributions of individual factors to wealth-related inequalities impacting childhood vaccination dropout among children aged 12 to 35 months in remote and underserved settings in Ethiopia by use of a decomposition approach. The overall CnI was –0.179, and it was statistically significant (*P* <0.01), which confirmed the concentration of vaccination dropout among the lowest wealth strata.

Our study also showed that wealth index contributed about 50% of the total inequalities in vaccination dropout. This finding is consistent with studies conducted in low- and middle-income countries,^9^^,^^14^^,^^17^^,^^32^^,^^33^ where lower dropout rates and improved vaccination coverage are pro-rich. In Benin, an even higher CnI was reported among children from rich homes, emphasizing higher concentration in childhood vaccination among children of the rich.^34^ A study conducted in sub-Saharan Africa showed that countries with lower vaccination coverage had higher inequalities, suggesting pro-rich coverage. As a result, they concluded that increasing coverage addresses inequalities.^35^ However, a study across Gambia, Kyrgyz Republic, and Namibia showed the opposite, where receipt of all basic vaccinations was disproportionately concentrated among children from poor households. Although the difference in vaccination completion rates between rural and urban areas mainly contributed to the concentration of vaccination among the poor in the Gambia and Namibia, it was chiefly household wealth that did so in the Kyrgyz Republic.^5^^,^^6^ Furthermore, in Nigeria and Guinea, vaccination dropout was more concentrated among children from the “most advantaged” backgrounds than among their counterparts.^10^

The other major contributors to inequalities in vaccination dropout were whether mothers had at least once seen an SBA (33.6%) and ANC contacts (5.7%) when they were pregnant. Mothers who had seen an SBA and had four or more ANC contacts were concentrated among people of higher socioeconomic status with a lower vaccination dropout. This might be because contacts with an SBA provide information about childbirth, newborn care, and immunization.^21^^,^^36^

The CnI for mothers who had lower educational status was negative, showing that these mothers were concentrated among people of the lowest wealth strata. The contributions of education might be due to the role that it plays in improving maternal health literacy and health care utilization behavior. Mothers with higher levels of education are also more likely to adhere to the immunization schedule, with a lower dropout rate.^4^^,^^18^^,^^31^^,^^32^^,^^37^^–^^41^

Children who were residing in rural areas and who were living in areas where it takes more than 30 minutes to get to the nearest health facility were also concentrated among people of the lowest wealth strata. Place of residence also explained –16.2% of the inequality in vaccination dropout. This finding is consistent with the findings of similar studies.^3^^,^^7^^,^^14^^,^^26^^,^^31^^,^^42^^,^^43^ The inequality related to place of residence could partly be explained by challenges faced in rural areas due to less developed health infrastructure and fewer skilled providers. In rural areas, traveling long distances to get to health facilities is another reason for low basic vaccination coverage and high dropout. In addition, lack of transportation access, travel costs, and waiting time pose critical challenges for mothers to take their child for vaccination.^18^^,^^44^ Vaccines are transported and stored in cold chain because they can easily be damaged by high temperatures.^45^ However, health facilities in rural areas face a shortage of electric power supply to keep the cold chain equipment working, which could impede provision of vaccination services as a result of stockout of vaccines.^46^ Vaccine and supply stockouts also contributed to the problem.^47^ Nonfunctionality of refrigerators for vaccine storage could also affect the provision of vaccination services.^48^ In contrast, a study in Ghana showed that children in rural areas were more likely to complete the required vaccinations, which might be attributed to the expansion of primary health care in rural Ghana.^49^

Ethiopia can drastically reduce the inequality in child vaccination coverage by strengthening women’s utilization of health care services and the accessibility of health facilities in rural kebeles. Vietnam achieved almost equal vaccination coverage among the rich and poor in 2014 by increasing disbursement of immunization staff across all areas of the country to ensure completely free vaccination for both the rich and the poor,^50^ whereas South Africa in 2016, Ghana in 2014, Burundi in 2016/2017, and Uganda in 2016 achieved little or no inequality in vaccination through increased vaccination coverage.^35^

This study had a number of noteworthy strengths. Despite the presence of active conflicts in certain study areas, the research team was able to gather data in those areas. Information gathered from various sources was used to determine childhood vaccination status. Vaccination cards, medical records, and maternal recall were used to ascertain vaccination dropout. The results were validated and verified by triangulating these three sources. Acquiring high-quality data was made possible through the use of digital tools and skilled data collectors. The decomposition of the contributing factors that drive socioeconomic inequalities in childhood vaccination dropout is of paramount importance for policymakers to address the socioeconomic disparities that exist around childhood vaccination coverage and dropout in Ethiopia.

The study’s limitations included its incapacity to draw conclusions about causality and its vulnerability to biases such as nonresponse bias and recall bias, which are directly linked to the study design, i.e., cross-sectional. For IDPs and refugee settings, if an immunization card was not available, the only way to determine a child’s immunization status was through the mothers’/caregivers’ self-reports/recalls. In this case, misclassification may result from mothers/caregivers who lack vaccination cards, forgetting their children’s vaccination status.

## CONCLUSION

Predictor variables that contributed the most to socioeconomic inequalities in vaccination dropout were wealth index, place of residence, SBA, and availability of a health facility in the kebele. Policymakers in Ethiopia need to address the pro-rich inequality in childhood vaccination by strengthening women’s utilization of health care services and the accessibility of health facilities in rural kebeles.
